# Characterization of Novel *Przondovirus* Phage Adeo Infecting *Klebsiella pneumoniae* of the K39 Capsular Type

**DOI:** 10.3390/v17121600

**Published:** 2025-12-10

**Authors:** Nadezhda V. Kolupaeva, Peter V. Evseev, Victoria A. Avdeeva, Angelika A. Sizova, Natalia E. Suzina, Nikolay V. Volozhantsev, Anastasia V. Popova

**Affiliations:** 1 State Research Center for Applied Microbiology and Biotechnology, City District Serpukhov, Moscow Region, 142279 Obolensk, Russia; nadin.9830@mail.ru (N.V.K.); fbun.avdeeva@yandex.ru (V.A.A.); sizova1508@gmail.com (A.A.S.); nikvol@obolensk.org (N.V.V.); 2 Pirogov Russian National Research Medical University, 117997 Moscow, Russia; petevseev@gmail.com; 3 Skryabin Institute of Biochemistry and Physiology of Microorganisms, Federal Research Center “Pushchino Scientific Center for Biological Research of the Russian Academy of Sciences”, 142290 Pushchino, Russia; suzina_nataliya@rambler.ru

**Keywords:** *Klebsiella pneumoniae*, bacteriophage, genomic analysis, tailspike protein, depolymerase, capsular type

## Abstract

*Klebsiella pneumoniae* is one of the most significant nosocomial pathogens and an important cause of human infections worldwide. The microorganism is capable of producing different capsular polysaccharides (CPSs), which are the primary receptors for capsule-specific *K. pneumoniae* bacteriophages encoding tailspike proteins (TSPs) with polysaccharide-degrading activity. In this study, the novel virulent *Przondovirus* phage Adeo was isolated and characterized. The phage was able to infect *K. pneumoniae* strain with a K39 capsular polysaccharide structure. The morphology, biological properties, stability, and genomic organization of Adeo were studied. Comparative genomic and phylogenetic analyses were performed to establish the relationship between the phage and other bacterial viruses. The gene encoding TSP Adeo_gp48 was identified and cloned. Recombinant depolymerase lacking the *N*-terminal part was expressed, purified, and formed an opaque zone of CPS depolymerization on the K39 *K. pneumoniae* bacterial lawns. The structural and phylogenetic similarities of Adeo’s TSP to other phage-encoded depolymerases were discussed.

## 1. Introduction

*Klebsiella pneumoniae* is a Gram-negative, nonmotile, facultative anaerobic, encapsulated bacterium belonging to the family *Enterobacteriaceae* [1]. It can be a component of the human microbiota, but at the same time, it is considered a significant nosocomial pathogen that causes various hospital-acquired infections [2,3]. The microorganism is often associated with pneumonia, meningitis, respiratory tract, bloodstream, urinary tract, and surgical site infections, especially in hospitalized immunocompromised patients, patients with underlying chronic illnesses, newborns, and elderly patients [4,5]. Because of the strong potential of *K. pneumoniae* to form an antibiotic-resistant phenotype, it commonly causes infection outbreaks worldwide [4,6]. Thus, *K. pneumoniae* is ranked by the World Health Organization (WHO) among the critical priority microorganisms for developing of new antibacterial agents [7]. In this regard, the application of lytic bacteriophages (phages), viruses that specifically infect and lyse bacterial cells, may be one of the approaches to control the spread of multidrug-resistant *K. pneumoniae* strains.

*K. pneumoniae* produces numerous variants of surface structures, capsular polysaccharides (CPSs), which differ from each other by the contest and number of monosaccharides in the repeating polysaccharide units and linkages formed between them [8]. CPSs are one of the most significant virulence factors [9,10] and play an important role in bacterial cell survival [11] and immune response evasion [12,13]. To date, more than 180 capsule biosynthesis gene clusters (K loci, KL) have been bioinformatically recognized in different *K. pneumoniae* genomes [14]. Many lytic phages infecting *K. pneumoniae* contain genes encoding structural proteins with polysaccharide-depolymerizing activities or tailspike proteins (TSPs), which facilitate the attachment and adsorption of a phage to a bacterial host cell belonging to corresponding capsular type (K type) by degrading CPS with a certain structure [15,16,17].

In this study, we characterized the novel phage Adeo, which exhibits lytic activity against *K. pneumoniae* of the K39 capsule type. The K39 CPS structure was established in 1987 by depolymerization of the CPS polymer with endo-β-d-glucosidase of phage φ39, which was not characterized [18]. During the past five years, the isolation of *K. pneumoniae* strain carrying KL39 was reported from hospitalized and intensive care unit patients of different medical centers in Moscow and Saint Petersburg (Russia) [19,20,21].

The genus *Przondovirus*, to which Adeo belongs, constitutes the large group of phages infecting *Klebsiella* spp. and comprises 229 bacterial viruses with genomes that have been deposited in the National Center for Biotechnology Information (NCBI) GenBank by September 2025. Genomes of Przondoviruses encode tailspike proteins (TSPs) responsible for the ability of the bacterial viruses to infect *K. pneumoniae* strains with a certain CPS structure. In this group, viruses specific to K1 [22,23], K2 [23,24,25,26,27,28], K3 [29], K5, K8 [30], K10 [27], K11 [31], K21 [29,32], K30 [30], K47 [33,34], K56 [35], K57 [22,23,27,36]; K64 [36,37,38,39,40,41,42,43,44], K69 [30], K102 [27], K106 [27,45], and KN1, KN3, KN4 [35] *K. pneumoniae* capsular types (K types) have already been described. Phage Adeo is the first bacterial virus studied that is specific to *K. pneumoniae* with a K39 CPS structure among all Przondoviruses.

The study of phages that can infect different K types contributes to our understanding of the diversity of *K. pneumoniae* bacterial viruses and to the development of strategies to combat infections caused by this microorganism.

## 2. Materials and Methods

### 2.1. Phage Isolation, Propagation, and Purification

Phage Adeo was isolated from a sewage sample collected in the Moscow region (Russia) in 2024 on a bacterial lawn of *K. pneumoniae* strain KPB-1434/16 obtained from the State Collection of Pathogenic Microorganisms and Cell Cultures (SCPM-Obolensk, Obolensk, Russia) (SCPM-Obolensk accession number: B-8048, GenBank accession number: JAFEHO000000000.1, capsular type K39). The sample was centrifuged at 6000× *g* for 20 min. The supernatant was filtered through 0.45-µm-pore-size membrane filter (Merck Millipore, Cork, Ireland). The filtrate was supplemented with an equal volume of LB medium and incubated overnight at 37 °C with shaking in the presence of growing *K. pneumoniae* strains belonging to different capsular types. A portion of chloroform (10% *v*/*v*) was then added, and the sample was centrifuged at 6000× *g* for 20 min. The supernatant was filtered through 0.45- and 0.22 µm-pore-size membrane filters (Merck Millipore, Cork, Ireland) to remove bacterial debris.

A single plaque with a halo found on the lawn of *K. pneumoniae* KPB-1434/16 was picked up in the SM buffer (10 mM Tris-HCl, pH 7.5, 10 mM MgSO_4_, and 100 mM NaCl) and plaque-purified three times to obtain pure phage stock. The phage was propagated using *K. pneumoniae* KPB-1434/16 liquid culture (OD_600_ of 0.3–0.4) at a multiplicity of infection (MOI) of 0.1. The incubation was performed at 37 °C with shaking until lysis, and chloroform was then added. Bacterial debris was pelleted by centrifugation at 13,000× *g* for 5 min. The phage particles were precipitated by polyethylene glycol (PEG) 8000 (added to a final concentration of 10% *w*/*v*) and 500 mM NaCl for 24 h at 4 °C.

### 2.2. Determination of Phage and Depolymerase Specificity

The host specificity of phage Adeo was tested against 56 *K. pneumoniae* strains belonging to 19 different K types (K1, K2, K3, K10, K17, K20, K23, K24, K27, K28, K31, K39, K47, K48, K54, K57, K60, K62, and K64) (Table S1) using the double-layer method [46]. For this procedure, 300 µL of *K. pneumoniae* bacterial cultures grown in medium «GRM-broth» (8 g pancreatic hydrolysate of fish meal, 8 g dry enzymatic peptone, 4 g sodium chloride, SRCAMB, Obolensk, Moscow region, Russia) at 37 °C to an OD_600_ of 0.4 (~10^9^ CFU/mL) were mixed with 4 mL of soft agar (GRM broth supplemented with 0.6% agarose) and then plated onto the nutrient agar. Subsequently, the phage (~10^8^ PFU/mL) or purified recombinant depolymerase, and their several dilutions, were spotted on the soft agar lawns and incubated at 37 °C for 18–24 h. The efficiency of plating (EOP) was calculated as the ratio of the number of PFU per test strain to the number of PFU per host strain [47].

### 2.3. Phage Adsorption and One-Step Growth Experiments

For the adsorption assay, exponentially grown *K. pneumoniae* KPB-1434/16 bacterial cells were mixed with the phage Adeo at MOI = 0.001 and incubated at room temperature. A volume of 100 µL of samples was taken in 0, 1, 2, 3, 4, 5, 8, 10, 15, and 20 min and then mixed with 850 µL of SM buffer supplemented with 50 µL of chloroform. After centrifugation, the supernatants were titrated for further determination of unabsorbed phages at different time intervals by the plaque assay method. The adsorption constant was calculated according to Adams [46].

For the one-step growth experiments, 20 mL of host bacterial cells (OD_600_ of 0.4) was harvested by centrifugation (7000× *g*, 5 min, 4 °C) and resuspended in 0.5 mL GRM broth. Bacterial cells were infected with the phage at a MOI of 0.01. The phage was allowed to adsorb at 37 °C for 2 min. Then, the mixture was centrifuged at 10,000× *g* for 2 min to remove unabsorbed phage particles, and the pellet was resuspended in 20 mL of GRM broth. Samples were taken at 5-min intervals over a 2-h incubation period at 37 °C and immediately titrated.

### 2.4. Stability of Phage at Different Temperatures and pH Values

The thermal stability of the phage was tested over a period of 1 h at 8 °C, 24 °C, 37 °C, 42 °C, 56 °C, 70 °C, and 92 °C. Phage titer was calculated using the plaque assay. To determine the pH stability, the phage was incubated at 37 °C for 1 h in SM buffers with pH values ranging from 3.2 to 12. A titer of 2.4 × 10^10^ PFU/mL was chosen for the experiments.

### 2.5. Electron Microscopy

For negative staining, phage Adeo (10^9^ PFU/mL) was placed onto grids coated with formvar film and then treated with a 0.3% aqueous solution of uranyl acetate (pH 4.0) after drying. The samples were examined with a JEM-1400 (JEOL, Tokyo, Japan) transmission electron microscope at an accelerating voltage of 80 kV.

### 2.6. DNA Isolation and Sequencing

Phage DNA was isolated using a standard phenol-chloroform method [48] after incubating of the sample in 0.5% SDS and 50 µg/mL proteinase K at 65 °C for 20 min. The GenoLab M platform (GeneMind Biosciences Co., Ltd., Shenzhen, China) with SG GM Plus (SESANA, Ltd., Moscow, Russia) and GenoLab Sequencing set V2.0 (FCM 300 cycles) (GeneMind Biosciences Co., Ltd., Shenzhen, China) were used for phage genome sequencing. The generated reads were assembled de novo into a single contig using SPAdes v.3.13 [49] with default parameters. The genome sequence of phage Adeo was deposited to GenBank under accession number OR855706.

### 2.7. Analysis of the Phage Genome and Proteins

Multiple nucleotide and protein sequence alignments were generated using MAFFT v7.48 with the L-INS-i algorithm under default options [50]. The resulting alignments served as input for phylogenetic inference in IQ-TREE v2.2.5, where ModelFinder selected the best-fit substitution model for each dataset and nodal support was assessed with 1000 ultrafast bootstrap replicates using the parameter set “-m TEST-bb 1000” [51]. The intergenomic relatedness among phages was assessed using VIRIDIC v1.1 under default settings, which computes pairwise identities through BLASTN-based comparisons and applies the recommended clustering thresholds [52]. Protein structures were predicted using AlphaFold 3 under default settings [53]. The top-ranked models were retained for downstream analyses, visualized in PyMOL v2.5.4 (Schrödinger Inc., New York, NY, USA), and used for pairwise superpositions. Structural similarity searches were performed with the DALI software (https://ekhidna2.biocenter.helsinki.fi/dali/, accessed on 1 October 2025), and the DALI Z-score was used as the primary metric of fold similarity, with RMSD and alignment length examined where appropriate [54,55]. Putative antibiotic resistance genes were queried using BLAST (https://blast.ncbi.nlm.nih.gov/Blast.cgi, accessed on 1 October 2025) against the Comprehensive Antibiotic Resistance Database (CARD) v3.2.7 [56]. Virulence factors were searched using the VFDB 2025 database [57]. Protein functions were inferred with HHpred under default options against the pdb70_from_mmcif_2023-06-18, pfam-a v35, and uniprot_sprot_vir70_Nov_2021 databases [58]. BLAST [59] was used to perform additional homology searches against a custom database constructed from genomic sequences retrieved from the GenBank PHG database. All software was run with the indicated versions, and unless specified otherwise, default parameters were applied.

### 2.8. Cloning, Expression and Purification of Recombinant Proteins

The DNA fragment of phage tailspike depolymerase (gp48; GenBank accession WQZ01644) lacking *N*-terminal domain was amplified by PCR using oligonucleotide primers 5′-GAACAGATTGGTGGTGTATCCGCCATGTCTTTACAGCA-3′ and 5′-TACCTAAGCTTGTCTTTAGTGAATTGCCTCCCACCCTG-3′ and cloned in the linearized pET SUMO vector (Thermo Fisher Scientific Inc., Waltham, MA, USA) by Gibson Assembly (New England Biolabs, Ipswich, MA, USA).

Expression vector was transformed into chemically competent *Escherichia coli* BL21(DE3) cells. Protein expression was performed in LB medium supplemented with 50 mg/L kanamycin. Transformed cells were grown at 37 °C until the optical density reached the value of 0.4 at 600 nm. The medium was cooled to the temperature of 16 °C, followed by the induction of expression by the addition of isopropyl-1-thio-β-D-galactopyranoside (IPTG) to a final concentration of 1 mM. After further incubation at 16 °C overnight (approximately 16 h), the cells were harvested by centrifugation at 3700× *g* for 20 min, at 4 °C. The cell pellets were resuspended in 1/50th of the original cell volume in buffer A (20 mM Tris, pH 7.5, 0.3 M NaCl, 5% glycerol). After that, resuspended cell pellets, divided into several aliquots, were lysed by sonication (three cycles with a 10-s on-time and a 20-s off-time) using Misonix S-4000-010 Ultrasonic Liquid Processor (Misonix Inc., Farmingdale, NY, USA). The cell debris was removed by centrifugation at 16,000× *g* for 30 min, 4 °C. The supernatants were loaded onto nickel Ni^2+^-charged GE HisTrap column (GE Healthcare Life Sciences, Marlborough, MA, USA) equilibrated with buffer A, and eluted with a 0–300 mM imidazole linear gradient in buffer A. The fractions containing the target proteins were pulled together and set up at 4 °C for the His-tag overnight digestion with SUMO-protease at a protease/protein ratio of 1/100 (*w*/*w*). This reaction mixture was simultaneously dialyzed against 20 mM Tris pH 7.5, 150 mM NaCl, 0.5 mM DTT (dithiothreitol) buffer to remove the His-SUMO expression tag. Protein samples after digestion were applied to the His-Trap column as before. A flow through was concentrated with ultrafiltration devices (molecular weight cutoff of 10,000) and stored in the same buffer at 4 °C.

### 2.9. Phage Infection Inhibition Assay

Adeo infection inhibition by purified recombinant TSP Adeo_gp48 was performed according to the published procedure [60]. A titer of 1.0  ×  10^5^ PFU/mL for the phage was chosen for the competition experiments. *K. pneumoniae* KPB-1434/16 was grown in LB medium at 37 °C to an OD_600_ of 0.3. Subsequently, TSP Adeo_gp48 was added to a 100-μL aliquot of the cell culture to a final concentration of 0.5 mg/mL and incubated for 20 min at 37 °C. One hundred microliter aliquots of the *K. pneumoniae* host cells without anything and with bovine serum albumin (BSA) to a final concentration of 0.5 mg/mL incubated for 20 min at 37 °C served as controls. After incubation, several dilutions of phage Adeo and 4 mL of soft agar were added to the mixtures and plated onto the nutrient agar. Plates were incubated overnight at 37 °C and the number of lysis plaques was determined. The experiments were performed in triplicate. GraphPad Prism 8.0 software (GraphPad Software, Inc., La Jolla, CA, USA) was used for statistical analysis and graphical presentation of the results.

## 3. Results

### 3.1. Morphological Characteristics and Infection Parameters of Phage Adeo

**Figure 1:**
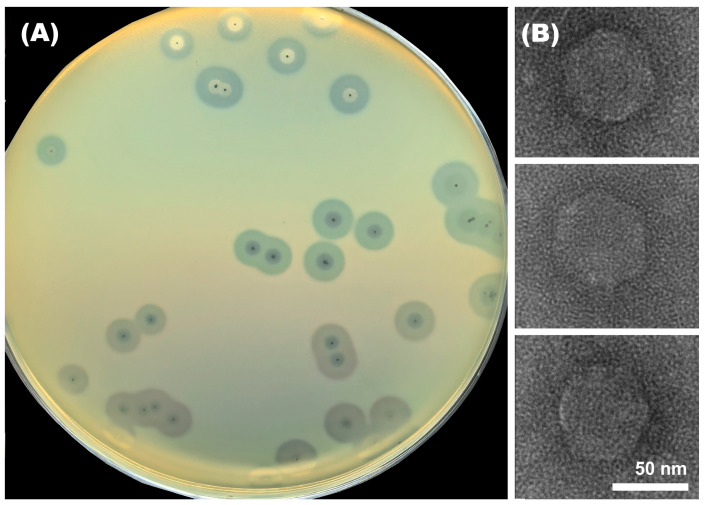
(**A**) Adeo plaques with opaque haloes on *K. pneumoniae* KPB-1434/16. (**B**) Transmission electron micrographs of different phage Adeo particles. Staining with 0.3% uranyl acetate. The scale bar is 50 nm.

Phage Adeo was isolated from a sewage sample by the enrichment procedure using *K. pneumoniae* strain KPB-1434/16 of the K39 type (GenBank accession: JAFEHO000000000.1) as the host bacterium. On the lawn of the host strain, Adeo produces clear plaques (about 2–5 mm in diameter) with opaque haloes (Figure 1A) formed as a result of the depolymerizing activity of phage tailspikes toward the CPS layer surrounding bacterial cells. Transmission electron microscopy of negatively stained phage particles revealed that Adeo has an icosahedral head of approximately 60 nm in diameter and a short noncontractile tail of up to 10 nm (Figure 1B).

The host specificity of the phage was determined using a collection of *K. pneumoniae* strains (*n* = 56) belonging to 19 different K types (Table S1). Among all tested strains, Adeo was able to infect only the fourteen strains with K39 CPS structure (Table S1). By comparing the titers of the phage on the thirteen *K. pneumoniae* strains of K39 types to the titer on the host strain *K. pneumoniae* KPB-1434/16, the EOP values for these strains varied from 0.1 to 0.91 (Table S1).

**Figure 2:**
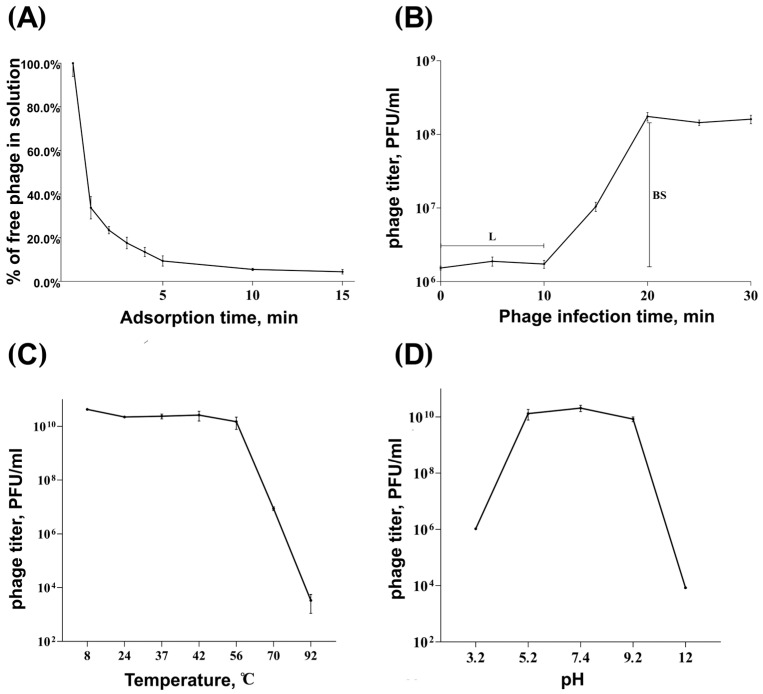
Infection analysis and phage Adeo stability. Adsorption assay (**A**) and one-step growth curve (**B**) of phage Adeo on *K. pneumoniae* KPB-1434/16 with the indication of estimated burst size (BS) and latent period (L). (**C**) Activity of phage Adeo in a range of different temperatures during 1 h of incubation. (**D**) Stability of phage Adeo in various pH conditions during 1 h of incubation. Results are presented as means and standard deviations from three independent experiments. PFU: plaque-forming units.

The parameters of the infection process were investigated in adsorption efficiency and one-step growth experiments. It was determined that above 60% of phage particles adsorbed to *K. pneumoniae* KPB-1434/16 cells within 1 min, and more than 90% within 5 min (Figure 2A). Phage Adeo exhibited an adsorption constant of 4.7 × 10^−10^ mL/min for the host strain for a period of 5 min. The one-step growth experiments revealed that Adeo had a latent period of 10 min, and the burst size was approximately 100 particles per infected cell (Figure 2B). As shown in Figure 2C, phage Adeo retained its infectivity in a temperature range of 8–56 °C, with a partial loss of infectivity at 70 °C and a very pronounced decrease its activity at 92 °C. The optimal pH values for phage Adeo were between 5.0 and 9.0, with a partial loss of activity at pH values of 3.2 or 12 (Figure 2D).

### 3.2. Analysis of Phage Genome and Phage-Derived Proteins

#### 3.2.1. General Characterization of the Adeo Genome

**Figure 3:**
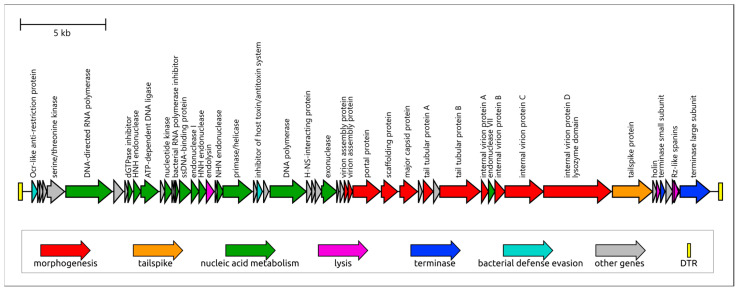
Genetic map of the *Klebsiella* phage Adeo. Predicted genes are depicted as arrows oriented in the transcription direction. Functional annotations and predicted gene products are indicated by labels and legends. The scale bar denotes nucleotide positions along the genome. DTR: direct terminal repeats.

Phage Adeo possesses a linear double-stranded DNA (dsDNA) genome (GenBank accession number: OR855706) of 41,389 bp, comprising 60 predicted genes, all encoded on the forward strand (Figure 3). The genomic G + C content was 52.9%, which was slightly lower than that of its host, *K. pneumoniae* KPB-1434/16 (56.8%). No tRNA genes were identified. The genome harbors 182 bp direct terminal repeats. No genes encoding toxins or other virulence factors and no antibiotic-resistance determinants, were detected.

#### 3.2.2. Genome Structure and Functional Modules

**Figure 4:**
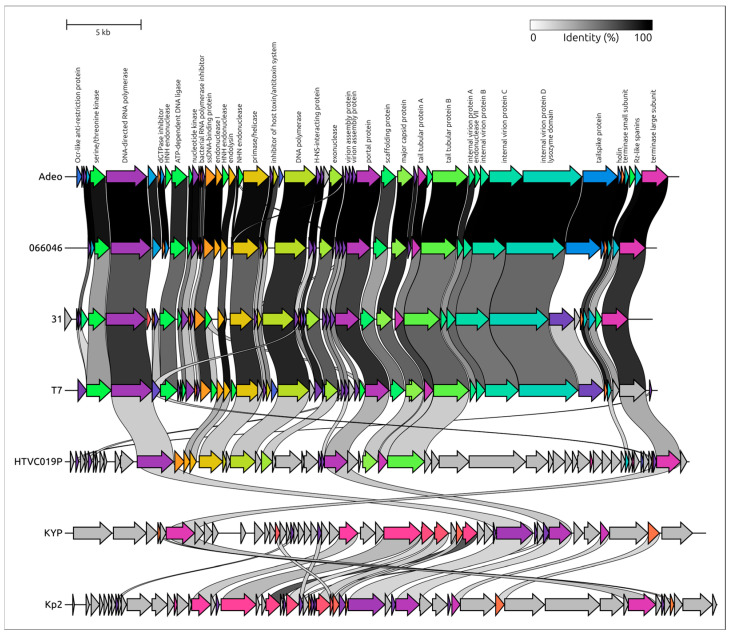
Comparative genomic map of *Klebsiella* phage Adeo, *Klebsiella* phage 06646, *Escherichia* phage T7, *Pelagibacter* phage HTVC019P, and *Klebsiella* phage Kp2. Arrows indicate predicted genes and their transcriptional orientation. The grayscale scale bar indicates gene identity with a 25% cutoff. Genes encoding similar proteins are rendered in the same colors across genomes, whereas non-homologous proteins are shown in gray.

The phage genome has a modular organization reminiscent of T7-like phages *sensu lato*, which are currently classified within the order *Autographivirales* and the family *Autotranscriptaviridae*, comprising only the subfamily *Studiervirinae* (https://ictv.global/taxonomy, accessed on 20 October 2025) (Figure 3). Closely related phages identified by whole-genome BLAST include members of the genus *Przondovirus* infecting *Klebsiella pneumoniae*, in particular those annotated in NCBI GenBank as *Klebsiella* phage 066046 (ICTV species *Przondovirus* 066046), vB_Kpl_K48PH164C1 (*Przondovirus* K48PH164C1), and *Klebsiella* phage vB_Kpl_K58PH129C2 (*Przondovirus* K58PH129C2). Together with these phages and other *Studiervirinae* phages, Adeo shares a characteristic gene order that differentiates it from some non-*Studiervirinae* phages (e.g., *Klebsiella* phage KYP, subfamily *Melnykvirinae*; Figure 4). This organization includes a T7-like DNA-dependent RNA polymerase (RNAP)—a hallmark of the order *Autographivirales*—located in the early region of the genome [61,62,63]. Interestingly, a similar gene order was observed in the more distantly related *Pelagibacter* phage HTVC019P [64], which may represent an early-diverged group within *Autographivirales* (Figure 4). This pattern suggests that the ancestor of *Studiervirinae* phages likely possessed a broadly similar genomic architecture. The Adeo genome contains a replication module that includes a T7-like family A DNA polymerase, a primase/helicase, nucleases, and a DNA ligase. As well as the general genome organization, the order and composition of the replication genes are reminiscent of those in *Escherichia* phage T7 and other *Autographivirales Studiervirinae* phages. In this module, we identified an inserted endolysin gene encoding an N-acetylmuramoyl-L-alanine amidase homologous to the T7 lysozyme (gp3.5). In phage T7, this enzyme is a Zn^2+^-dependent amidase that cleaves the MurNAc–L-Ala bond of peptidoglycan and, together with the holin (gene 17.5) and spanins, mediates host–cell lysis; T7 lysozyme also binds and inhibits T7 RNAP to tune transcription timing [65]. These homologies suggest a similar enzymatic mechanism for Adeo endolysin. The packaging module encodes small and large terminase subunits, typical of *Heunggongvirae* phages. The morphogenesis block includes a T7-like podoviral portal protein (PP), a major capsid protein (MCP), four internal virion proteins that appear less conserved than MCP and PP, two tubular proteins, and accessory genes involved in virion assembly.

Furthermore, genome comparisons revealed clear similarities across most genes among the analyzed *Studiervirinae* phages, including Adeo and *Escherichia* phage T7, with lower conservation observed for nonstructural genes and internal virion proteins, although robust homology is retained for RNAP and other replication genes. However, the predicted tailspike protein (TSP) does not show significant homology to receptor-binding proteins of related *Studiervirinae* phages outside the genus *Przondovirus*, which is apparently explained by differences in the adsorption mechanism and ability of the phage to recognize of CPS of a certain structure. Another notable feature of the Adeo genome is the presence of bacterial defense-evasion genes. In particular, it encodes a putative Ocr-like protein that can function as an anti-restriction factor by mimicking DNA and inhibiting the type I DNA restriction–modification system [66] and possibly the BREX defense system [67]. The Ocr-like protein is conserved in other *Studiervirinae* viruses, including T7, although its sequence appears to be less conserved than its structure and function. Gene prediction also indicates two overlapping spanin genes, inner and outer spanin, a feature not previously annotated in most related *Przondovirus* phages and, to the authors’ knowledge, not discussed earlier, possibly due to the non-obvious positioning of these genes. This brings Adeo closer to phage T7, although the latter carries embedded spanin genes [68]. Finally, unlike some closely related *Studiervirinae* phages, including *Klebsiella* phage 06646, the Adeo genome contains a putative NHN endonuclease gene located upstream of the DNA ligase gene. A BLAST search revealed a mosaic pattern of presence and absence of this gene upstream of the DNA ligase among different phages within the subfamily *Studiervirinae*.

#### 3.2.3. Taxonomy and Signature Genes Phylogeny

The taxonomic assignment of phage Adeo was performed according to common ICTV requirements [69]. Intergenomic comparisons were conducted using two datasets: a 117-genome set comprising ICTV-classified representatives of the genus *Przondovirus* and a 50-genome set containing diverse representatives of the order *Autographivirales* (Figures S1 and S2). The search revealed the highest intergenomic similarity of 89.1% to *Klebsiella* phage 06646 (ICTV species *Przondovirus* 066046), followed by 83.5% to *Klebsiella* phage FZ12 (*Przondovirus FZ12*) and 82.9% to *Klebsiella* phage vB_Kpl_K58PH129C2 (*Przondovirus K58PH129C2*). Using the 95% and 70% species and genus delineation cutoff, respectively, these values unambiguously assign phage Adeo to a new species, *Przondovirus Adeo*, within the genus *Przondovirus* [69]. Proteome-based ViPtree phylogeny supports these conclusions, placing phage Adeo within the *Przondovirus* clade (Figure S3).

At larger genetic distances, intergenomic comparisons indicate the relatedness of *Przondovirus* phages to other genera within the subfamily *Studiervirinae* (approximately 17–59%), particularly *Benllochvirus* (*Klebsiella* phage cp31), *Apdecimavirus_AP10* (*Yersinia* phage vB_YenP_AP10), *Eapunavirus Eap1* (*Enterobacter* phage phiEap-1), and *Yuanmingyuanvirus NJ2* (*Enterobacter* phage NJ2). Notably, the intergenomic similarity between *Przondovirus* representatives and the *Wuhanvirus* representative *Pasteurella* phage vB_PmuP_PS07, recently (2024) assigned by the ICTV to the subfamily *Studiervirinae*, was the lowest (approximately 16–17%), whereas the similarity values between *Przondovirus* phages and non-*Wuhanvirus Studiervirinae* genera were approximately 32–59%.

**Figure 5:**
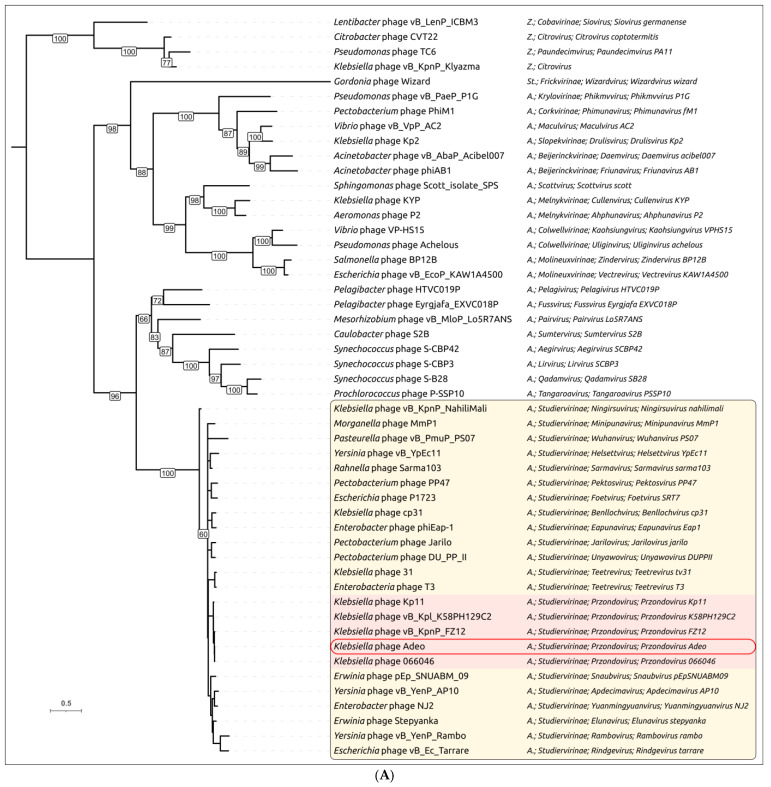

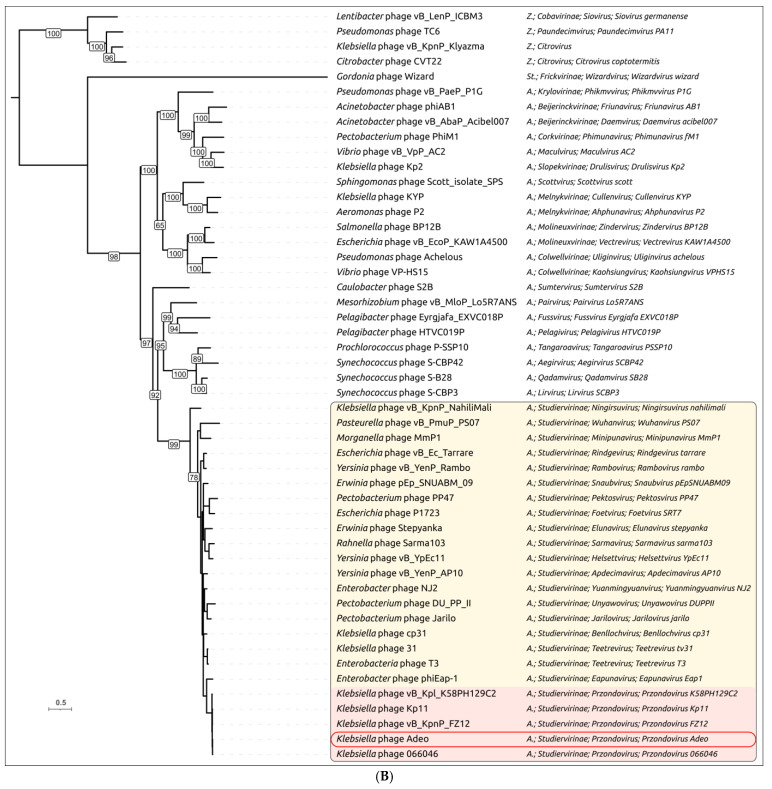
Maximum-likelihood phylogenies of the major capsid protein (**A**) and the large terminase subunit (**B**). Bootstrap support values are shown at nodes; the scale bar indicates substitutions per site. Colored areas highlight *Studiervirinae*; the *Przondovirus* group is shaded light red; Adeo is outlined in red. Clades with bootstrap support < 50 are shown as polytomies. The ICTV taxonomy is shown to the right. The abbreviations “A.”, “St.”, and “Z” denote *Autographivirales*, *Stackebrandtviridae*, and *Zobellviridae*, respectively.

Phylogenetic analysis based on signature gene phylogenies was conducted using the amino acid sequences of the MCP (Figure 5A) and the terminase large subunit (TLS) (Figure 5B). Sequences were extracted from the genomes of 50 phages representing the order *Autographivirales*, including phages used in the intergenomic similarity calculations above, with additional *Autographivirales*-related phages from the families *Stackebrandtviridae* and *Zobellviridae* included as outgroups. This analysis generally supports the results described above. Phylogenies of MCP and TLS consistently place *Klebsiella* phage Adeo within *Autographivirales*, family *Autotranscriptaviridae*, subfamily *Studiervirinae*, genus *Przondovirus*. In the MCP tree, Adeo forms a well-supported sister relationship with *Klebsiella* phage 066046. This pair clusters with *Klebsiella Przondovirus* phages vB_KpnP_FZ12, Kp11, and KpK1_KSBPH129C2. The encompassing clade includes diverse *Enterobacterales* phages. The TLS phylogeny reproduced the same topology around Adeo and its closest neighbors, and the broader *Przondovirus* assemblage was recovered with strong support and similar intergeneric relationships. Overall congruence between the structural (MCP) and packaging (TLS) markers indicates vertical inheritance of these modules in Adeo and coevolution of these proteins, which may reflect a lower gene-content flux in lytic phages [70,71]. However, the placement of the temperate *Gordonia* phage Wizard in the MCP tree within one of two distinct clades that both contain *Autographivirales* raises questions about the origin and early evolution of *Autographivirales* and *Stackebrandtviridae* viruses. Furthermore, in the TLS tree, *Pasteurella* phage vB_PmuP_PS07 falls outside *Przondovirus*, grouping with *Morganella* phage MmP1 and its allies. Distant cyanophages and *Pelagibacter* phages form earlier-diverging branches that precede the *Studiervirinae* clade.

#### 3.2.4. Tailspike Protein Analysis

**Figure 6:**
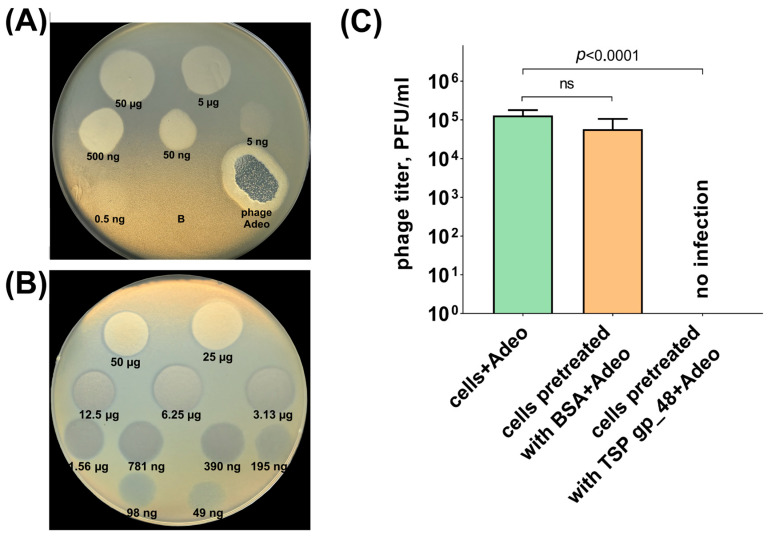
Spot test with serial 10-fold (**A**) and 2-fold (**B**) titration of purified recombinant TSP Adeo_gp48 on *K. pneumoniae* KPB-1434/16 lawn after 16 h of incubation. B—buffer for storage of the protein as a negative control. Phage Adeo—a spot formed by the phage Adeo (10 µL from a phage preparation with the titer of 10^8^ PFU/mL). (**C**) Phage Adeo infection inhibition by TSP Adeo_gp48. From left to right, phage titers were observed on the bacterial lawns after the treatment of *K. pneumoniae* KPB-1434/16 cells with phage Adeo only, after preincubated of cell cultures with BSA (as a negative control, at a final concentration of 0.5 mg/mL), and with purified TSP Adeo_gp48 (at a final concentration of 0.5 mg/mL), followed by phage Adeo treatment. Significance was determined using the *t*-test. *p*: *p*-value, ns: not significant.

The genome of the phage Adeo encodes only one TSP with depolymerizing activity (Adeo_gp48, GenBank accession: WQZ01644) that determines specificity to *K. pneumoniae* with K39 CPS structure. Recombinant Adeo-derived depolymerase lacking the *N*-terminal domain was specific and formed an opaque halo (zone of depolymerization) on the bacterial lawn of *K. pneumoniae* KPB-1434/16 (Figure 6A,B) and on the lawns of the other *K. pneumoniae* of K39 type (*n* = 14, listed in Table S1) studied.

Phage infection inhibition or competition experiments have been performed to demonstrate that TSP Adeo_gp48 is responsible for the initial step of the phage Adeo-host cells interaction (Figure 6C). *K. pneumoniae* KPB-1434/16 host preincubated with purified recombinant protein Adeo_gp48 and with BSA in the negative-control experiment were mixed with several phage dilutions and plated on agar plates. After overnight incubation, phage titer was measured. It was shown that coincubation with Adeo_gp48 resulted in the *K. pneumoniae* KPB-1434/16 host cells becoming nonsusceptible to infection by phage Adeo. In other words, the addition of purified depolymerase Adeo_gp48 to the host cell at the chosen concentration (0.5 mg/mL) completely inhibited plaque formation. This means that the depolymerase effectively degraded the K39 CPS layer surrounding the *K. pneumoniae* KPB-1434/16 host cells and, consequently, the specific phage Adeo carrying TSPs with K39 polysaccharide-degrading activity could not specifically recognize the primary receptor, the K39 CPS polymer, and bind to the cell surface. In a negative-control experiment, host bacterial cells were pretreated with BSA (at the same concentration); after that, no significant differences in phage titers compared with the control without any protein were shown. This means that coincubation of host cells with BSA does not affect the K39 polymer capsule, and the phage could effectively bind to the cell at the initial step of infection.

**Figure 7:**
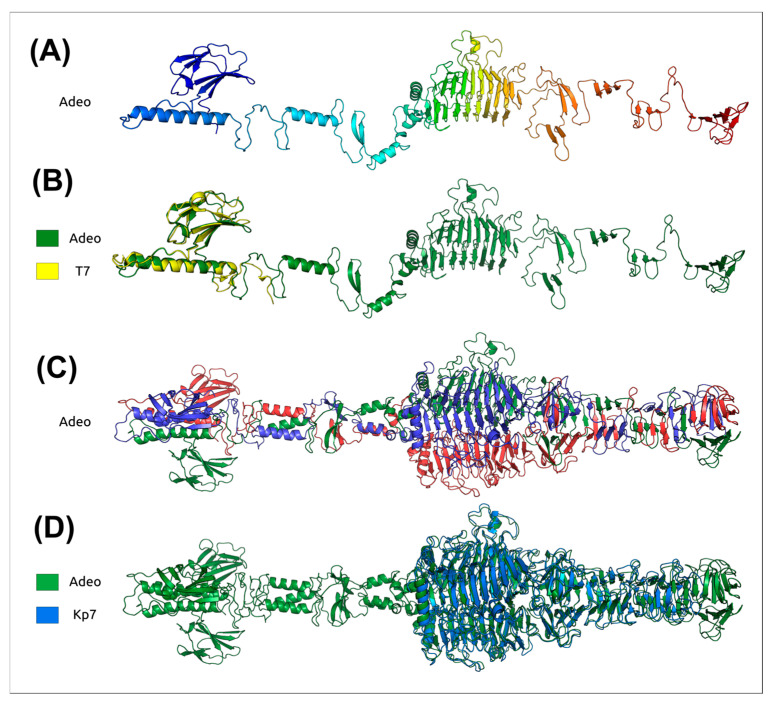
Structural analysis of the TSP Adeo_gp48. (**A**) TSP Adeo_gp48 model colored from *N*-terminus to *C*-terminus. (**B**) the *N*-terminal region (residues 6–141) of the *Escherichia* phage T7 tail fiber protein (PDB ID: 9JYZ) superposed on the Adeo’s TSP model; RMSD = 1.183 Å (Adeo, green; T7, yellow). (**C**) Adeo’s TSP trimer model, each subunit is depicted in a different color. (**D**) *C*-terminal depolymerase domain of the *Klebsiella* phage Kp7 depolymerase (PDB ID: 7Y5S) superposed on the TSP Adeo_gp48 trimer model; RMSD = 1.141 Å (Adeo, green; Kp7, blue).

HMM-based analysis and structural modeling of Adeo’s TSP indicated its bipartite architecture, which included an *N*-terminal assembly/adapter module (*N*-terminus) and CPS-recognizing/degrading part or *C*-terminal polysaccharide-depolymerase module (*C*-terminus) (Figure 7). Structure-based superposition of the Adeo_gp48 *N*-terminus onto the homologous region (residues 6–141) of the *Escherichia* phage T7 tail fiber protein (PDB ID: 9JYZ [72]) yielded RMSD = 1.183 Å, indicating near-identity of the fold and supporting a conserved podoviral tail-attachment module. Conversely, the Adeo_gp48 *C*-terminus does not align to T7 but superposes onto the β-helix depolymerase domain of *Klebsiella* phage Kp7 (PDB ID: 7Y5S, https://www.rcsb.org/structure/7Y5S, accessed on 28 October 2015) with RMSD = 1.141 Å, indicating that Adeo carries a *Klebsiella*-type CPS depolymerase fused to a T7-like *N*-terminal adapter. These observations match the canonical functional division in podoviral tailspikes which imply conserved *N*-terminal trimerization/tail-docking and a variable, β-helix depolymerase that determines capsule specificity in *Klebsiella* phages [15,16,17].

**Figure 8:**
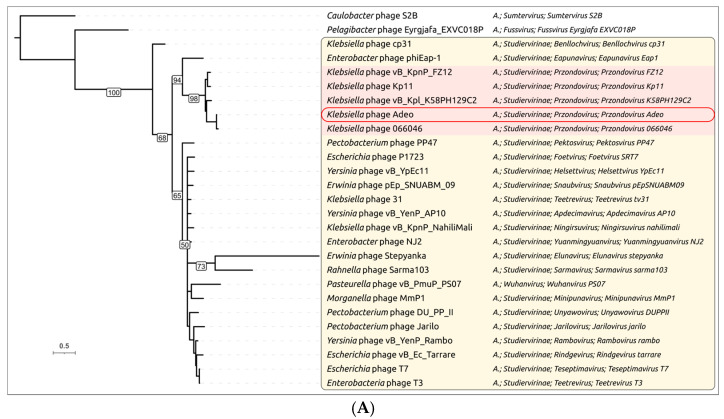

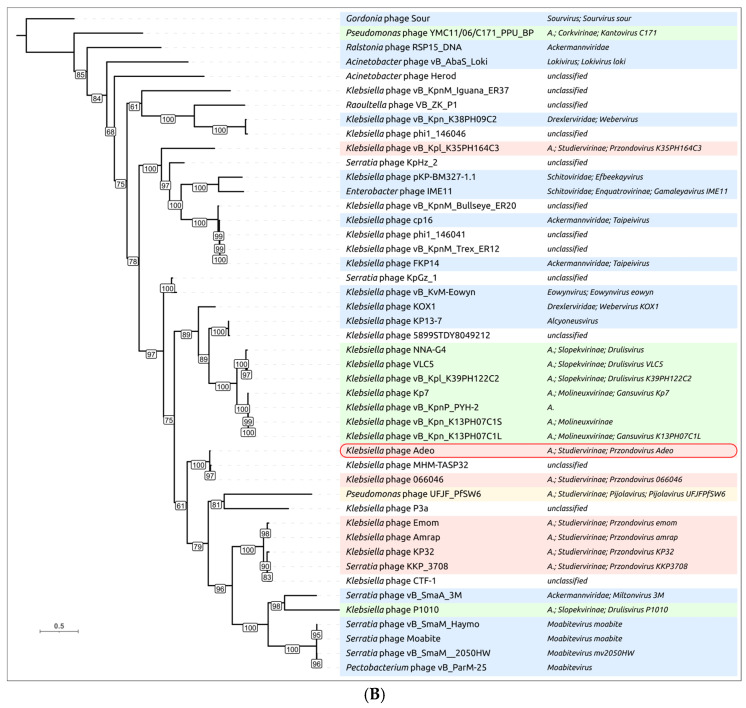
Maximum-likelihood trees of the *N*-terminal (**A**) and *C*-terminal region (**B**) of TSPs from representative phages. Bootstrap values are shown at nodes; scale bar, substitutions per site. Colored areas highlight *Studiervirinae* (light red), not-*Studiervirinae* phages (green) and other phage groups (blue) classified by ICTV or in NCBI annotations; Adeo is outlined. Clades with bootstrap support values < 50 are shown as polytomies. The ICTV or NCBI taxonomy is shown on the right. The abbreviation “A.” stands for *Autographivirales*.

Phylogenetic analysis using the closest phage sequences found by BLAST searches over the GenBank PHG database confirmed distinct evolutionary trajectories of the *N*- and *C*-terminal parts of Adeo’s TSP. In the *N*-terminus tree (Figure 8A), Adeo_gp48 clusters within the *Autographivirales Autotranscriptaviridae Studiervirinae Przondovirus* group alongside *Klebsiella Przondovirus* phages, including Kp11, KSBPH129C2, FZ12, and 066046. The tree preserves the expected relationships among these *Enterobacteriaceae* podoviruses. Together with the T7-like structural match (RMSD = 1.183 Å to 9JYZ), this topology indicates the vertical inheritance of the *N*-terminal domain within *Przondovirus* and the absence of recent domain replacement at the *N*-end. The *C*-terminus tree (Figure 8B) shows a markedly different picture. In this tree, Adeo_gp48 no longer groups exclusively with *Przondovirus*; instead, it falls inside a mixed *Klebsiella* depolymerase clade comprising phages from multiple taxa (e.g., *Przondovirus*, *Drulisvirus*/*Ganusvirus*-like groups, and even distantly related phages of the *Ackermannviridae* lineages). This crosstaxon clustering mirrors the convergent exchange of CPS-depolymerase domains among *Klebsiella* phages that attack the same or related capsule types [17,73].

The CPS-recognizing/degrading part of Adeo_gp48 shares a high percentage of amino acid similarity with the proteins encoded in Przondovirus 066046 (QOV07395) and unclassified *Klebsiella* phage MHM-TASP32 (XUJ68802). The similarity of amino acid sequences of these proteins indicates that they most likely interact specifically with CPS of the same structure, namely K39 CPS.

The close structural match of Adeo’s *C*-terminus to Kp7 depolymerase (RMSD = 1.141 Å to 7Y5S) is congruent with this placement and supports the assignment of Adeo_gp48 *C*-terminus to the right-handed β-helix lyase/hydrolase family, which is typical for *Klebsiella* capsule depolymerases. Comparing the two single-gene phylogenies demonstrates domain-wise incongruence. The *N*-terminus follows the virion-assembly lineage signal of *Przondovirus* (consistent with core virion genes such as MCP and TLS), whereas the *C*-terminus tracks substrate (capsule) specificity, grouping with depolymerases from *Klebsiella* phages of different taxonomy and origin. This discordance indicates a modular evolutionary history of Adeo’s TSP, with a conserved T7-like adapter inherited vertically within *Przondovirus* and a laterally acquired *Klebsiella* CPS-depolymerase module at the *C*-end.

## 4. Discussion

Phage Adeo is the first Przondovirus studied that is specific to *K. pneumoniae* belonging to the K39 type. The phage formed clear plaques with haloes, indicating the presence of structural proteins or tailspikes with depolymerizing activity. Recombinant Adeo-derived enzyme also formed zone of depolymerization on the bacterial lawn of *K. pneumoniae* KPB-1434/16 and effectively degraded K39 CPS of this strain inhibiting phage Adeo-host binding. The phage was characterized by rapid adsorption, large burst size, high stability, and the absence of genes encoding toxins, virulence factors, and antibiotic-resistance determinants in the genome. This indicates its potential for further practical usage as a candidate for controlling nosocomial infections caused by *K. pneumoniae* of the K39 type.

Genomic analysis indicated that Adeo belongs to *Autographivirales*, family *Autotranscriptaviridae*, subfamily *Studiervirinae*, genus *Przondovirus*, and shows a T7-like genomic layout: the early region contains the single-subunit RNA polymerase, the replication, packaging, and morphogenesis modules follow the canonical order for T7-like podoviruses. Intergenomic similarity and proteome phylogeny place Adeo within *Przondovirus* and support the status of a new species. MCP and TLS trees recover near-identical neighborhood for Adeo and resolve a compact *Klebsiella Przondovirus* cluster. Congruent placements of these two hallmark virion proteins indicate their vertical inheritance, which is consistent with a lower gene-content flux expected for strictly lytic lineages [70,71] and explains the high bootstrap support around the Adeo node in both trees. Comparisons with non-*Studiervirinae* podoviruses refine these conclusions: while *Klebsiella Przondovirus* phages form a coherent group across structural markers, *Pasteurella* phage vB_PmuP_PS07 falls outside this group in the terminase tree and supports the boundary of the Adeo clade. Distant cyanophages and pelagiphages form long-branched outgroups and do not affect Adeo placement. The observation that a T7-like early organization also appears in distant *Autographivirales* such as HTVC019P suggests that the common ancestor of *Autographivirales* already possessed a similar early transcription–replication architecture. Taken together, the single-gene analyses of signature markers robustly support the assignment of Adeo to the genus *Przondovirus* and clarify details of early evolution of *Autographivirales*.

Tailspike analysis revealed a clear division of evolutionary histories within a single protein. The *N*-terminus of the TSP Adeo_48 matches the T7 tail assembly adapter with near-identity of fold. The *N*-terminal tree clusters Adeo with *Klebsiella Przondovirus* phages and almost mirrors this part of the MCP and TLS topologies. This pattern indicates vertical inheritance of the adapter module within *Przondovirus*. The module likely mediates trimerization and docking to the phage particle and does not constrain receptor specificity [74,75,76]. The *C*-terminus shows a different signal. It aligns with the β-helix depolymerase domain of *Klebsiella* phage Kp7 and groups in a mixed *Klebsiella* depolymerase clade that spans several taxa. Such clustering reflects the exchange of polysaccharide-depolymerase domains among *Klebsiella* phages targeting the same or related capsules [73,76]. These observations explain the joint behavior of Adeo in single-gene phylogenies: markers tied to virion architecture, such as MCP, TLS, and the *N*-terminus of the tailspike, track the taxonomic backbone of *Przondovirus*, whereas the *C*-terminus of the tailspike follows capsule structure rather than phage taxonomy. Domain-wise incongruence indicates modular evolution with frequent lateral replacement of receptor-binding and catalytic regions [77,78].

## Data Availability

The genome sequence of phage Adeo is available in GenBank under accession number OR855706.

## Supplementary Materials

The following supporting information can be downloaded at: https://www.mdpi.com/article/10.3390/v17121600/s1, Figure S1: VIRIDIC intergenomic similarity clustered heatmap using 117 classified *Przondovirus* phages; Figure S2: VIRIDIC intergenomic similarity clustered heatmap for 117 genomes comprising ICTV-classified representatives of the genus *Przondovirus*; Figure S3: Proteome-based phylogenomic tree generated with ViPtree from genome-wide tBLASTx similarities; Table S1: The host specificity and lytic activity of phage Adeo.

## Abbreviations

The following abbreviations are used in this manuscript:

bp	base pair
CFU	Colony forming Unit
PFU	Plaque Forming Unit
MOI	Multiplicity of Infection
EOP	Efficiency of Plating
ICTV	International Committee on Taxonomy of Viruses
