# Supplementary material for: Characterization of Novel Przondovirus Phage Adeo Infecting Klebsiella pneumoniae of the K39 Capsular Type
Source: Viruses. 2025 Dec 10;17(12):1600. doi: 10.3390/v17121600 (PMC12737672; doi:10.3390/v17121600)
Supplement: Supplementary file 1 [file viruses-17-01600-s001.zip › viruses-4006684-supplementary.pdf]

**Table S1. The host specificity and lytic activity of phage Adeo.**

| No. | K type | Designation of *K. pneumoniae* strain | SCPM-Obolensk accession number | Lysis | EOP |
|---|---|---|---|---|---|
| 1 | **1** | B-2580 | SCPM-O-B-7852 | - | - |
| 2 | | B-475 | SCPM-O-B-7854 | - | - |
| 3 | | KPI-1683 | SCPM-O-B-7938 | - | - |
| 4 | | I-261 | SCPM-O-B-7850 | - | - |
| 5 | | S-73 | SCPM-O-B-7841 | - | - |
| 6 | **2** | B-4010 | SCPM-O-B-7846 | - | - |
| 7 | | B-755 | SCPM-O-B-8392 | - | - |
| 8 | | i-1627 | SCPM-O-B-7849 | - | - |
| 9 | | i-1748 | SCPM-O-B-8040 | - | - |
| 10 | | i-6208 | SCPM-O-B-7666 | - | - |
| 11 | **3** | ATCC 13883 | SCPM-O-B-4811 | - | - |
| 12 | **10** | KPB 498 | SCPM-O-B-10168 | - | - |
| 13 | | KPB 711 | SCPM-O-B-10172 | - | - |
| 14 | | KPB536-14 | SCPM-O-B-B-8658 | - | - |
| 15 | | B-944 | SCPM-O-B-7882 | - | - |
| 16 | **17** | KPS4208 | SCPM-O-B-15808 | - | - |
| 17 | **20** | M9 | SCPM-O-B-7749 | - | - |
| 18 | | KPS82148 | SCPM-O-B-16460 | - | - |
| 19 | **23** | B-2304K/15 | SCPM-O-B-8047 | - | - |
| 20 | | KPi4275 | SCPM-O-B-7935 | - | - |
| 21 | | KPS6483 | SCPM-O-B-16478 | - | - |
| 22 | **24** | KPB1674 | SCPM-O-B-7934 | - | - |
| 23 | **27** | KPB941 | SCPM-O-B-10173 | - | - |
| 24 | **28** | KPB591 | SCPM-O-B-15726 | - | - |
| 25 | **31** | KPB-1128-1 | SCPM-O-B-10175 | - | - |
| 26 | **39** | **KPB-1434/16** | **SCPM-O-B-8048** | + | 1 |
| 27 | | KR100 | SCPM-O-B-9458 | + | 0,41 |
| 28 | | KR126/21 | SCPM-O-B-9950 | + | 0,66 |
| 29 | | KR69m/22 | SCPM-O-B-10240 | + | 0,27 |
| 30 | | KR 190m/22 | SCPM-O-B-10227 | + | 0,18 |
| 31 | | KR 380m/22 | SCPM-O-B-10226 | + | 0,31 |
| 32 | | KR255p/22 | SCPM-O-B-10351 | + | 0,1 |
| 33 | | C22543k/21 | SCPM-O-B-10392 | + | 0,75 |
| 34 | | C7088-21 | SCPM-O-B-10666 | + | 0,66 |
| 35 | | KP5044 | SCPM-O-B-10914 | + | 0,15 |
| 36 | | AU Pr.5957 04_08_23 | SCPM-O-B-11437 | + | 0,5 |
| 37 | | 207-2023 | SCPM-O-B-11605 | + | 0,91 |
| 38 | | KPS8329 | SCPM-O-B-15817 | + | 0,62 |
| 39 | | KPS2894 | SCPM-O-B-17174 | + | 0,21 |
| 40 | **47** | KPB-1667 | SCPM-O-B-8043 | - | - |
| 41 | **48** | KPS78055 | SCPM-O-B-16462 | - | - |
| 42 | | KPS78054 | SCPM-O-B-16468 | - | - |
| 43 | **54** | ATCC12657 | SCPM-O-B-7887 | - | - |
| 44 | **57** | KPB-542/15 | SCPM-O-B-8042 | - | - |
| 45 | | KPB-612-1 | SCPM-O-B-7879 | - | - |
| 46 | | KPB-690 | SCPM-O-B-7874 | - | - |
| 47 | | B-811 | SCPM-O-B-7707 | - | - |
| 48 | | KPS3995 | SCPM-O-B-15811 | - | - |
| 49 | **60** | KPB-54/14 | SCPM-O-B-8041 | - | - |
| 50 | **62** | KPB1493-2 | SCPM-O-B-8710 | - | - |

| | | | | | |
|---|---|---|---|---|---|
| 51 | | B-1759 | SCPM-O-B-7667 | - | - |
| 52 | | B-420 | SCPM-O-B-10167 | - | - |
| 53 | 64 | KPB-417/16 | SCPM-O-B-7954 | - | - |
| 54 | | B-958/14 | SCPM-O-B-7810 | - | - |
| 55 | | KPS6123 | SCPM-O-B-17181 | - | - |
| 56 | | KPS7939 | SCPM-O-B-15814 | - | - |

SCPM: State Collection of Pathogenic Microorganisms and Cell Cultures

EOP: efficiency of plating

The host strain for phage Adeo is highlighted in red
