# Supplementary figures and images for: Characterization of Novel Przondovirus Phage Adeo Infecting Klebsiella pneumoniae of the K39 Capsular Type
Source: Viruses. 2025 Dec 10;17(12):1600. doi: 10.3390/v17121600 (PMC12737672; doi:10.3390/v17121600)

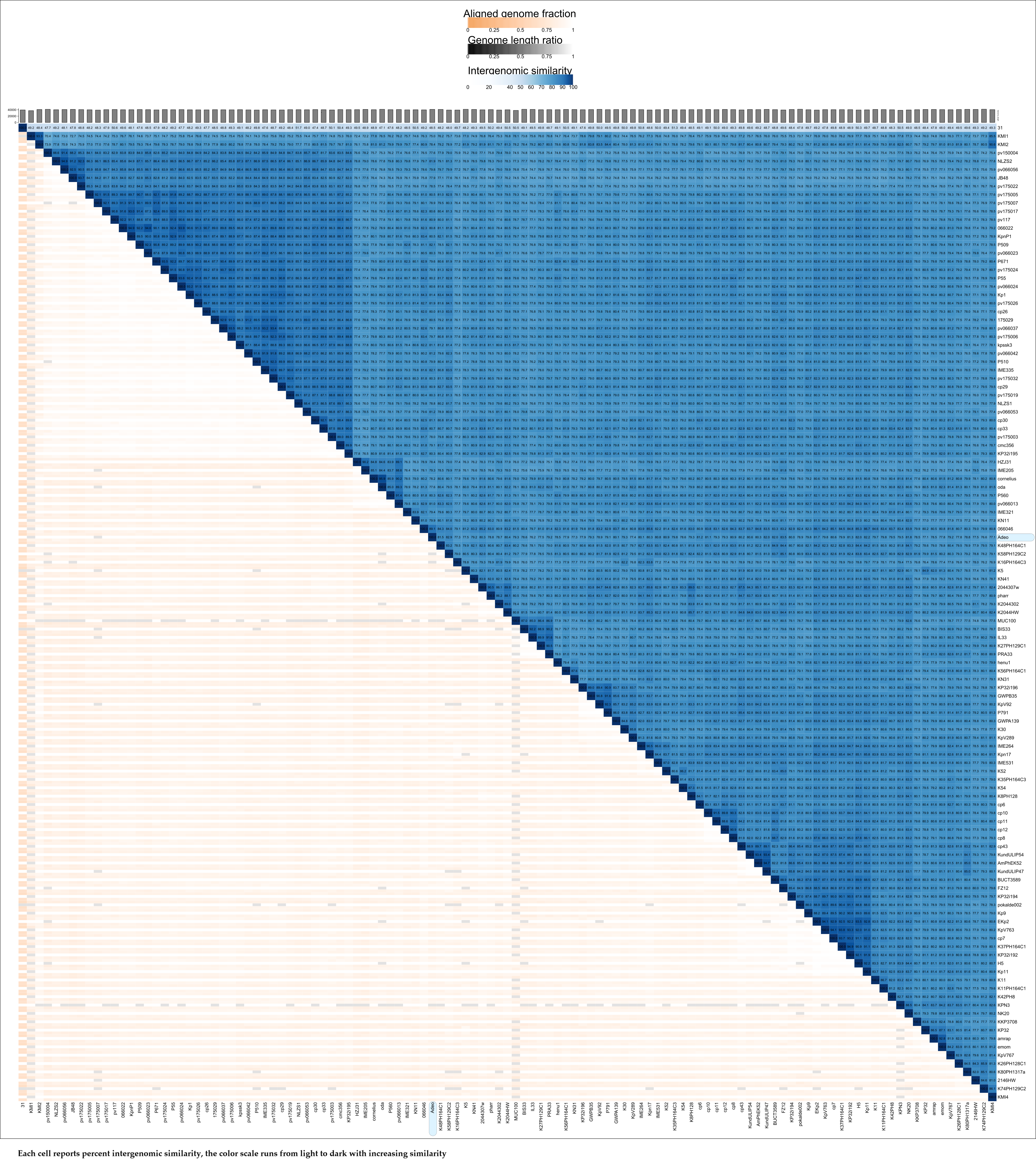

Supplement: Supplementary file 1 [file viruses-17-01600-s001.zip › Figure S1_VIRIDIC intergenomic similarity clustered heatmap using 117 classified Przondovirus phages.png]

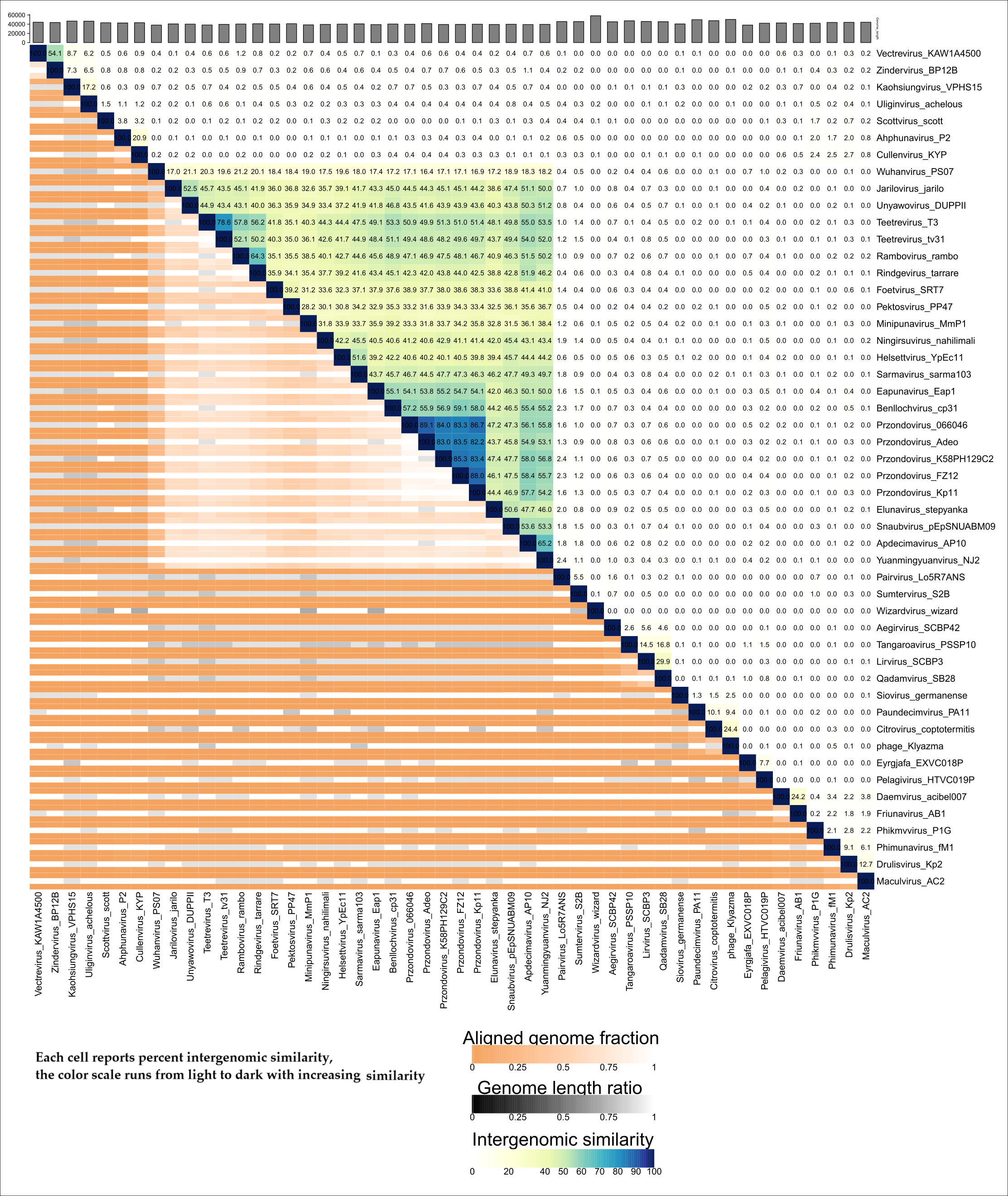

Supplement: Supplementary file 1 [file viruses-17-01600-s001.zip › Figure S2_VIRIDIC intergenomic similarity clustered heatmap for 117 genomes comprising ICTV-classified representatives of the genus Przondovirus.png]

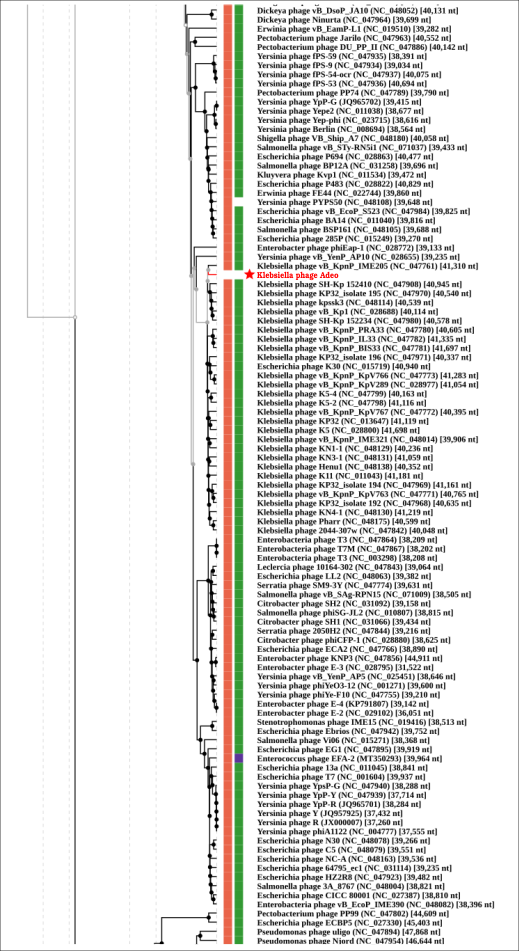

Supplement: Supplementary file 1 [file viruses-17-01600-s001.zip › Figure S3_Proteome-based phylogenomic tree generated with ViPtree from genome-wide tBLASTx similarities.png]
